# Optimal Background Color for Head-Starting Northern River Terrapins (*Batagur baska* Gray, 1831)

**DOI:** 10.3390/ani10020207

**Published:** 2020-01-26

**Authors:** Suthep Jualaong, Karun Thongprajukaew, Santi Ninwat, Natchapong Petchrit, Suwandee Khwanmaung, Wattana Wattanakul, Thana Tantipiriyakij, Hirun Kanghae

**Affiliations:** 1 Department of Marine and Coastal Resources, Marine and Coastal Resources Research and Development Center, Lower Gulf of Thailand, Songkhla 90100, Thailand; sutep.emcor@hotmail.com (S.J.); ninwat@hotmail.com (S.N.); 2 Department of Applied Science, Faculty of Science, Prince of Songkla University, Songkhla 90112, Thailand; 3 Satun Inland Aquaculture Research and Development Center, Satun 91110, Thailand; natch7930@gmail.com (N.P.); if-satuninland@hotmail.com (S.K.); 4 Department of Fisheries Technology, Faculty of Sciences and Fisheries Technology, Rajamangala University of Technology Srivijaya, Trang 92150, Thailand; wattanakul67@gmail.com; 5 Nai Yang Beach Resort and Spa, Phuket 83110, Thailand; gm@naiyangbeachresort.com; 6 Department of Marine and Coastal Resources, Phuket Marine Biological Center, Phuket 83000, Thailand; kanghae_h@hotmail.com

**Keywords:** carapace, feces, feed utilization, hematological parameter, wall color

## Abstract

**Simple Summary:**

Substrate color is an important physical condition affecting the lifestyle of reared animals. However, no prior data are available regarding northern river terrapins. In this study, the terrapins were reared in five types of colored tanks (transparent, green, red, blue, and black) for twelve weeks. Based on parameters assessing survival, growth, feed utilization, and health, the blue background is more suitable for rearing terrapins relative to other colored tanks. The findings from the current study could be applied to wall, pond, or aquaria decoration to support the head-starting programs of northern river terrapins before release to natural habitat, as well as in public displays, such as aquaria and zoos.

**Abstract:**

Background color has significant effects on the lifestyles of various animal species. In this study, the effects of wall background color on growth, feed utilization, specific activity of gastric and pancreatic enzymes in fecal samples, fecal thermal properties, carapace elemental profile, and hematological parameters were investigated in northern river terrapin (*Batagur baska* Gray, 1831) in order to optimize the head-starting conditions. The terrapins (70.13 ± 0.04 g initial weight) were completely randomized into five types of colored tanks (transparent, green, red, blue, and black) and reared for twelve weeks. At the end of the experiment, tank color had no effect on survival and growth performance, but had significant effects on feeding rate, feed conversion ratio, and protein efficiency ratio (*p* < 0.05). Terrapins reared with black, red, or blue backgrounds had the highest feed utilization among the treatments. Among these three groups, analysis of specific activity of gastric and pancreatic enzymes in fecal samples and fecal thermal properties suggested improved digestive functionality in terrapins reared with a blue background relative to the other treatments. Carapace elemental composition and hematological parameters indicated no negative effects on health status of the terrapins reared with this optimal treatment. Findings from the current study support the head-starting program of northern river terrapins before release to natural habitats, and could also be applied in aquaria or zoos for public display.

## 1. Introduction

The northern river terrapin (*Batagur baska* Gray, 1831) is a riverine turtle native to Southeast Asian countries (i.e. India, Bangladesh, Myanmar, Thailand, Cambodia, Malaysia, and Indonesia) [1]. It prefers freshwater habitats but also moves to brackish water or estuaries in the breeding season. This species is classified as critically endangered by the International Union for Conservation of Nature and Natural Resources (IUCN) and as a member of the Convention on International Trade in Endangered Species of Wild Fauna and Flora (CITES); it is also one of the 25 chelonians in urgent need of conservation action [2]. In Thailand, ex situ head-starting for release to natural habitat of this species has been conducted since 1983 by the Department of Fisheries [2].

Under captivity during head-starting, the conditions that affect terrapin quality and animal welfare can be improved. Substrate color is an important physical condition affecting the lifestyle of reared animals [3,4]. Therefore, animals from a wide range of taxonomic groups can change their body color based on their background over a range of spatial and temporal scales, since camouflage through color change has ecological significance [5], as well as significance for the physiological processes in response to background stimuli [6]. In turtles and tortoises, some evidence of color preferences has been reported [7,8,9,10], however the majority of such studies have been conducted in fish [11,12,13,14,15]; no prior data are available regarding terrapins. 

The background color interacts with light intensity and color, potentially causing stress and using up energy available from food digestion. The determination of digestive enzyme activities in order to assess feed utilization is a reasonable approach [16,17]. For endangered species, investigations of specific activity of gastric and pancreatic enzymes in fecal samples and fecal thermal properties have been demonstrated to provide information about available nutrients in feces [18,19,20]. These approaches are non-lethal, non-invasive, and not in conflict with ethical standards. 

The objective of this study was to experimentally determine a near optimal background color for head-starting northern river terrapins to support the mission of the Department of Fisheries and Department of Marine and Coastal Resources, Thailand. Alternative colors were assessed based on growth performance, feed utilization (as well as specific activity of gastric and pancreatic enzymes in fecal samples and fecal thermal properties), carapace elemental profile, and hematological parameters. The findings from the current study could be directly applied in head-starting for this species, as well as for rearing in zoos or aquaria.

## 2. Materials and Methods

The terrapins (*B. baska*) were obtained from an ex situ head-starting program in Satun province, Thailand. The terrapins were acclimatized in a concrete pond (2 m width × 3.8 m length × 0.6 m height) for 2 weeks. They were fed to satiation twice daily (08:30 and 14:30) with floating feed pellets for tadpoles (32.50% crude protein). The natural photoperiod of 12 h light/12 h dark was performed during acclimatization. Water was added to obtain 10 cm depth from the bottom before the first feeding and it was 100% drained out at 15:00. The water quality parameters during the experiment were within the following ranges: pH 7.5−8.0, temperature 25–28 °C, dissolved oxygen 4.5–7.0 mg L^−1^, alkalinity 64–162 mg L^−1^, and hardness 55–70 mg L^−1^.

The terrapins (70.13 ± 0.04 g initial weight) were completely randomized into five types of colored tanks (transparent, green, red, blue, and black) and reared for twelve weeks. The aquaria dimensions were 45 cm width × 90 cm length × 45 cm height, with 10 cm water level. There were fifteen experimental units for five treatments and three replicates with four terrapins each. The conditions for rearing terrapins were as described for the acclimatization period. Growth parameters were recorded every other week. Feed utilization parameters were estimated from the difference between the number feed pellets that were offered and those that remained after feeding. At the end of the 12 week trial, the terrapins were fasted for 12 h prior to collecting the specimens (feces, carapace, and blood). The parameters relating to growth and feed utilization were calculated as described below:

Survival (%) = (Final terrapin number/initial terrapin number) × 100(1)

Body condition index (BCI, kg cm^−3^) = (Body weight (kg)/straight carapace length (cm)^3^) × 10^4^ (2)

Specific growth rate (SGR, % body weight day^−1^) = ((ln W_t_ − ln W_0_)/(t − t_0_)) × 100 (3)

where Wt = mean weight (g) at day t, W_0_ = mean weight (g) at day t_0_.

Feeding rate (FR, % body weight day^−1^) = C/((W_0_ + W_t_)/2)/t × 100 (4)

where C = daily feed consumption (g), W_0_ = initial body weight (g), W_t_ = final body weight (g), t = feeding duration (day).

Feed conversion ratio (FCR, g feed g gain^−1^) = Dry feed consumed (g)/wet weight gain (g) (5)

Protein efficiency ratio (PER, g gain g protein^−1^) = Wet weight gain (g)/protein intake (g) (6)

The fresh feces (*n* = 3 per treatment) were quickly collected by dip net and were carefully rinsed by cold distilled water to eliminate dirt. Cold distilled water was added (1: 5 *w*/*v*) and then the mixtures were homogenized using a microhomogenizer (THP−220; Omni International, Kennesaw GA, USA). The extraction of digestive enzymes in fecal samples was conducted as described by Kanghae et al. [18]. The quantification of protein in crude enzyme extract was determined using bovine serum albumin as protein standard [21]. The assay conditions (including pH and temperature) used were the optimal ones for determining the specific activity of gastric and pancreatic enzymes in fecal samples in green turtles (*Chelonia mydas*) [20]. The activities of pepsin (EC 3.4.23.1), trypsin (EC 3.4.21.4) and chymotrypsin (EC 3.4.21.1), amylase (EC 3.2.1.1), and lipase (EC 3.1.1.3) were assayed as described by Worthington [22], Rungruangsak-Torrissen et al. [23], Bernfeld [24], and Winkler and Stuckmann [25], respectively. 

Fresh feces (*n* = 3 per treatment) were prepared according to the method described by Kanghae et al. [26]. Three milligrams of dried feces were heated from 40 to 400 °C at a rate of 10 °C min^−1^ in an empty pan. Onset (T_o_), peak (T_p_) and conclusion (T_c_) temperatures, and enthalpy (∆H) were recorded using a differential scanning calorimeter (DSC7; Perkin Elmer, Waltham, MA, USA). The temperature range (T_c_–T_o_) and total ∆H (Σ∆H) are reported.

The carapace sampling and preparation (*n* = 3 per treatment) were conducted as described in Kanghae et al. [26]. The carapace elemental composition was carried out with a scanning electron microscope (Quanta 400; Field Electron and Ion Co., Ltd. (FEI), Brno, Czech Republic) equipped with an energy dispersive X-ray spectrometer (X-MAX, Oxford, UK). 

The blood samples (*n* = 3 pooled samples per treatment) were harvested with 1 mL syringes from the dorsal cervical sinus. Red (RBC) and white (WBC) blood cell counts, packed cell volume (hematocrit), and mean corpuscular volume (MCV) were determined based on the methods of Blaxhall and Daisley [27], Larsen and Snieszko [28], and Dacie and Lewis [29], respectively. Blood urea nitrogen (BUN), creatinine, alkaline phosphatase (ALP), and alanine transaminase (ALT) were determined using a commercial diagnostic kit (PZ Cormay S.A. Company, Lomianki, Poland). Serum cortisol was determined based on electrochemiluminescence immunoassay (National Healthcare Systems Co., Ltd., Songkhla, Thailand).

The data were treated with Microsoft Office Excel 2010 (Microsoft Corp., Washington, WASH., USA) and analyzed in Statistical Package for Social Science Version 14 (SPSS Inc., Chicago, IL, USA). One-way analysis of variance (ANOVA) was used, and the significance of differences between means were tested with Duncan’s multiple range test, with significance equated to *p* ˂ 0.05. 

## 3. Results 

### 3.1. Survival, Growth Performance, and Feed Utilization

No mortality was observed during the twelve-week experiment (Table 1). Growth performance (final body weight, straight carapace width, straight carapace length, BCI, and SGR) did not differ across the five treatments (*p* > 0.05). Terrapins reared with a red, blue, or black background exhibited superior feed utilization traits, as indicated by comparatively low FCR and high FR and PER among the alternative treatments. 

### 3.2. Specific Activity of Gastric and Pancreatic Enzymes in Fecal Samples 

**Figure 1:**
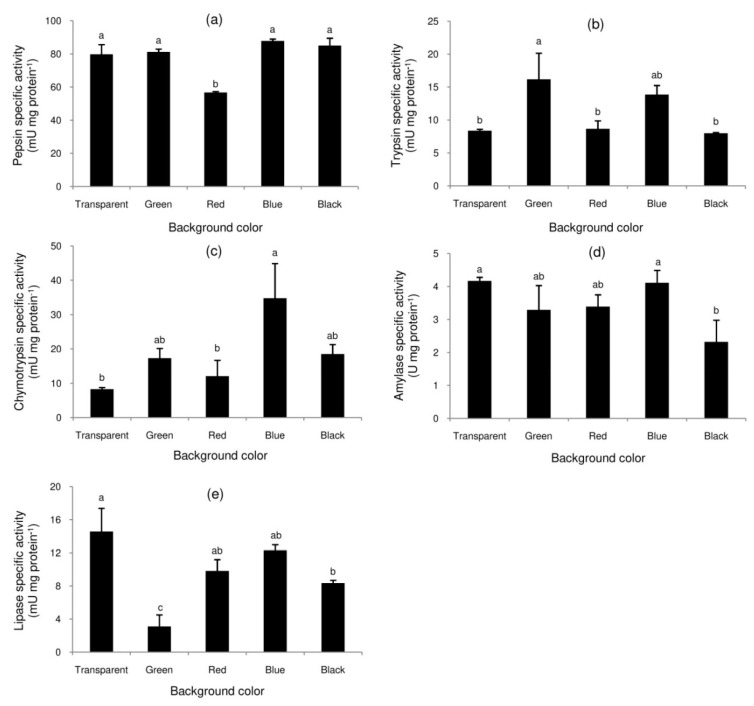
Specific activities of digestive enzymes in the feces of northern river terrapins reared with various background colors: pepsin (**a**), trypsin (**b**), chymotrypsin (**c**), amylase (**d**), and lipase (**e**). The feces were sampled at the end of the twelve-week experiment. Data are expressed as mean ± standard error of mean (*n* = 3 per treatment). Different superscripts indicate a significant difference (*p* < 0.05).

Terrapins reared with red background had the lowest pepsin-specific activity (Figure 1a). Specific activities of trypsin (Figure 1b) and chymotrypsin (Figure 1c) were relatively high in terrapins reared with green and blue backgrounds. The highest amylase-specific activity was observed in the terrapins reared with a transparent or blue background; its activity was the lowest with the black background (Figure 1d). Terrapins reared with transparent and green backgrounds had the highest and lowest lipase specific activities, respectively (Figure 1e).

### 3.3. Thermal Properties of Feces

A low temperature peak was not observed in the feces of terrapins reared with blue backgrounds (Table 2), while the thermal characteristics (T_o_, T_p_, T_c_, and T_c_–T_o_) were similar across the four remaining treatments. Terrapins reared with a black background had significantly higher (*p* < 0.05) ΔH than those reared with a transparent, green, or red background. Regarding the high temperature peak, no differences in T_o_, T_p_, or T_c_ were observed across the five treatments. The largest T_c_–T_o_ was observed in terrapins reared with a black background. The largest ΔH and ΣΔH were observed with a black or red background and the lowest was with a green or blue background.

### 3.4. Carapace Elemental Profiles

Eleven major elements were observed in the carapace of northern river terrapins (Table 3). Generally, the contents were similar across the five treatments, except for oxygen, sulfur, and calcium. Significantly decreased oxygen levels were observed in terrapins reared with a blue background. Based on sulfur and calcium accumulations, use of a transparent or black background was less suitable for rearing the terrapins than the other treatments.

### 3.5. Hematological Parameter

Hematocrit, plasma protein, BUN, creatinine, ALP, and ALT values did not differ across the five treatments (Table 4). RBC was significantly higher in terrapins reared with red or black background than with transparent or blue, and an intermediate value was recorded with green background; MCV values followed exactly the opposite pattern. WBC and lymphocytes were significantly higher in terrapins reared with the colored backgrounds than with a transparent tank. The lowest azurophil was observed in terrapins reared with the black background. The cortisol concentration was below the detection limit across the five treatments (< 0.02 μg dL^−1^).

## 4. Discussion

Although some evidence of color preferences was reported for turtles or tortoises [7,8,9,10], no significant differences in growth performance of terrapins were found across the five background colors in the current study. Similar observations were also reported for common carp (*Cyprinus carpio*) [15] and for convict cichlid (*Cichlasoma nigrofasciatum*) [12] reared with various background colors for 14 and 8 weeks, respectively. On the other hand, significant effects of background color on growth have been reported in white sea bream (*Diplodus sargus*) [14], goldfish (*Carassius auratus*) [13], and starlet (*Acipenser ruthenus*) [11]. These prior studies indicate that the growth responses of animals to background color are species-specific.

Significant improvement in feed utilization was clearly observed in terrapins reared with red, blue, or black backgrounds. In some aquatic species, especially in the early stages of development, improved feed consumption is associated with a sharp color contrast between feed and background [15,30]. Findings from the current study are inconsistent with prior reports, in that the feed color is dark brown and the terrapins were in the sub-yearling stage. It is possible that a neurohormonal mechanism, such as melanin-concentrating hormone (MCH), could enhance feed intake and utilization [14,31]. Visual observations of carapace and dorsal head skin in terrapins reared with dark backgrounds (blue or black) support this possibility. However, the color intensity of terrapins was not examined in the current study. In Kemp’s ridley sea turtles (*Lepidochelys kempii*), a pilot study indicated that basins, feed, and personnel dressed in red were preferred over other colors [8]. The authors believe that the behavior was primarily based on optical rather than olfactory sensations. Response in feed utilization of terrapins reared with a red, blue, or black background might be stimulated by various mechanisms, possibly with complicated interactions. Further investigations on feed utilization, including specific activity of gastric and pancreatic enzymes in fecal samples and thermal properties, might provide further useful data for rearing terrapins. In addition, it would be relevant to compare the effect of the background color in the composition of the microbiome, using the DNA extracted from fecal samples; it would be an interesting and novel approach to establish the effect of color in intestinal health.

Studying digestive enzymes provides information about the mechanisms of digestion and nutritional needs [32]. Data for comparison across the reptile species are scarce, and non-lethal (preferably non-invasive) techniques are needed for studies of endangered species, such as northern river terrapins. In shrimp, specific activity of pancreatic enzymes in fecal samples correlates well with the mid-gut enzymes, so that sampling of feces informs about the gut function [32]. However, this kind of comparative study can be performed with other non-endangered model turtle species, such as soft-shelled turtles (*Pelodiscus sinensis*), to collect more evidence prior to application in endangered species. Recently, the specific activities of gastric and pancreatic enzymes in fecal samples have been used as indicators for monitoring feed utilization in green turtles [18,19,20,26,33]. In the current study, the effects of background color on the specific activities of these digestive enzymes were clearly observed. Among the three observed proteolytic enzymes, pepsin is the most efficient digestive enzyme in the stomach, while trypsin and chymotrypsin play important roles in protein digestion in the intestine. The significantly decreased pepsin activity in terrapins reared with red background indicates a negative effect on the activity of gastric glands. Trypsin is responsible for approximately 40–50% of dietary protein digestion [34] and activates other zymogens, such as chymotrypsinogen, proelastase, procarboxypeptidase, as well as trypsinogen; therefore, up-regulation of trypsin and chymotrypsin in terrapins reared with green and blue backgrounds suggests superior protein digestion with these treatments. 

Maintaining the glucose level is important as it is the primary energy source for metabolic homeostasis [35]. Significantly decreased amylase activity in the current study suggests negative effects on carbohydrate utilization in terrapins reared with a black background. Lipids are a minor constituent in the experimental feed (3.66% on feed basis), while the major constituents are crude protein (32.50% as feed basis) and nitrogen-free extract (47.66% as feed basis). Increased lipase activity in terrapins reared with transparent background might be associated with a sparing effect due to poor protein digestion. However, negative effects were clearly observed in terrapins reared with a black or green background. Based on the overall enzyme activities observed, the optimal treatment was with a blue background, since it maintained or increased the specific activities of protein-, carbohydrate- and lipid-digesting enzymes. Some changes in light intensity could influence the pineal gland to stimulate enzyme activities [36]. Therefore, responses in digestive enzymes to varying light parameters have been reported for various species [17,37,38]. 

Major changes in thermal properties of fecal samples were observed across the five treatments. The low temperature peak indicates the presence of available nutrients, mainly proteins and carbohydrates, in the feces [20,26]. The absence of this peak with the blue background suggests complete digestion and absorption of nutrients along the alimentary tract. Regarding the second peak, the responses at these temperatures are associated with the change in amount of unavailable nutrients (probably fiber). Therefore, significantly decreased ΔH and ΣΔH indicate superior feed utilization with the optimal treatment. The difference in T_c_–T_o_ relates to the heterogeneity of digested molecules in the feces [39]. The significantly decreased difference in the terrapins reared with a blue background suggests the similarity of the digestion products, with the narrowest range for transformation being observed during heating.

Among the eleven chemical elements observed, significant differences were prominent in oxygen, sulfur, and calcium. Oxygen is the major element present in various chemical compounds [26]. Replacement of oxygen with sulfur and calcium was observed in terrapins reared in the optimal treatment conditions. In reptiles, abundance of the sulfur-containing amino acid cysteine in β-pleated keratin from carapace was observed [40]. Increased sulfur content might be linked to the amounts of keratins, which are a family of fibrous structural proteins [41]. Calcium is the predominant element in turtle carapaces [42]; it mainly forms as a carbonate that contributes to calcite-rich rock [43]. The comparatively high amounts of calcium and maintained phosphorus levels in terrapins reared with a red, green, or blue background might support a hard carapace and strong healthy bones [44,45]. However, rearing with a red or green background was not suitable because of negative effects on feed utilization. 

Hematological parameters are commonly referenced indicators of health status in captive animals. In the current study, some changes in hematological parameters associated with background colors were observed. Terrapins reared with the transparent or the optimal background had relatively low RBC counts and increased MCV. This indicates physiological acclimatization of terrapins by adjusting the RBC population and its average volume to the treatments. Suppressed innate immunity by lowering WBC [46] or lymphocytes [26] has been reported in animals reared under unsuitable conditions, while the population of azurophils is frequently associated with inflammation and infectious diseases [47]. These factors indicate negative effects on health status in terrapins reared with a transparent or black background in the current study. The concentration of plasma cortisol as an indicator of stress response was below the detection limit of <0.02 μg dL^−1^, which is also below that found in prior reports on healthy tortoises and turtles [48,49]. Generally, the steroid of most value in addressing the stress response in reptiles is corticosterone [50], while cortisol is found at very minimal levels in the blood and is not sensitive in most non-mammals [51]. Therefore, the current study could be improved upon by the use of a corticosterone assay.

## 5. Conclusions

Although no significant effects on survival and growth were observed across the five treatments of terrapins, feed utilization efficiency was significantly influenced by the background color. The terrapins reared with the blue background had improved digestive functionality with up-regulated main digestive enzymes, which were present in feces that also had a low remaining content of available nutrients. In addition, carapace elemental composition and hematological parameters indicated no negative effects on health status of the terrapins reared with the optimal treatment. Practically, the findings from the current study could be applied to wall, pond, or aquaria decoration to support the head-starting programs of northern river terrapins before release to natural habitats, as well as in public displays, such as aquaria and zoos. Overall, the study results help support the ex situ conservation of this species in Thailand. The transparent tank may be thought of as a control in the current study, however, in many ways it is the most unusual treatment for this type of bottom dwelling turtle. The darker tanks may be more similar to the actual environment where this species lives. Further studies comparing the blue background from the current study with the other darker tanks are of interest.

## Figures and Tables

**Table 1:** Survival, growth performance, and feed utilization of northern river terrapin reared with various background colors. The sampling represents the end of the twelve-week experiment.

Parameter	Background Color					*p*-Value
	Transparent	Green	Red	Blue	Black	
Survival (%)	100	100	100	100	100	–
FBW (g)	125.50 ± 11.65	148.46 ± 10.31	136.59 ± 5.37	136.14 ± 10.64	143.94 ± 6.16	0.495
SCW (cm)	8.99 ± 0.11	9.39 ± 0.07	9.31 ± 0.13	9.32 ± 0.21	9.42 ± 0.15	0.285
SCL (cm)	8.97 ± 0.33	9.42 ± 0.17	9.37 ± 0.17	9.40 ± 0.33	9.53 ± 0.09	0.531
BCI (kg cm^−1^)	1.73 ± 0.03	1.62 ± 0.03	1.66 ± 0.08	1.64 ± 0.04	1.66 ± 0.06	0.659
SGR (% BW day^−1^)	0.92 ± 0.16	1.05 ± 0.18	0.94 ± 0.10	1.04 ± 0.02	1.01 ± 0.11	0.702
FR (% BW day^−1^)	0.89 ± 0.02 ^c^	1.11 ± 0.06 ^a^	1.00 ± 0.01 ^ab^	1.03 ± 0.01 ^ab^	1.04 ± 0.02 ^ab^	0.018
FCR (g feed g gain^−1^)	1.46 ± 0.03 ^a^	1.21 ± 0.01 ^b^	1.06 ± 0.02 ^c^	1.03 ± 0.01 ^c^	1.08 ± 0.05 ^c^	0.001
PER (g gain g protein^−1^)	1.96 ± 0.04 ^c^	2.36 ± 0.01 ^b^	2.71 ± 0.04 ^a^	2.77 ± 0.01 ^a^	2.66 ± 0.11 ^a^	0.002

Note: FBW, final body weight; SCW, straight carapace width; SCL, straight carapace length; BCI, body condition index; SGR, specific growth rate; BW, body weight; FR, feeding rate; FCR, feed conversion ratio, PER, protein efficiency ratio. Data are expressed as mean ± standard error of mean (*n* = 12 per treatment). Differences between means were tested with Duncan’s multiple range test. Different superscripts in the same row indicate a significant difference (*p* < 0.05).

**Table 2:** The thermal transition characteristics of feces of northern river terrapins reared with various background colors. The sampling represents the end of the twelve-week experiment.

Thermal Parameter	Background Color					*p*-Value
	Transparent	Green	Red	Blue	Black	
***Peak 1***						
T_o_ (°C)	83.88 ± 0.76	81.67 ± 1.03	81.89 ± 0.25	ND	82.39 ± 0.74	0.495
T_p_ (°C)	92.17 ± 1.55	91.50 ± 0.93	90.44 ± 0.48	ND	89.93 ± 0.58	0.388
T_c_ (°C)	98.95 ± 2.69	93.29 ± 6.57	97.22 ± 1.33	ND	95.00 ± 1.57	0.724
T_c_–T_o_ (°C)	15.07 ± 2.02	14.52 ± 5.02	15.33 ± 1.29	ND	14.50 ± 2.07	0.745
ΔH (J g^−1^)	2.27 ± 0.65^b^	4.15 ± 0.51^b^	3.32 ± 0.81^b^	ND	8.11 ± 1.35^a^	0.020
***Peak 2***						
T_o_ (°C)	351.01 ± 2.29	351.12 ± 1.77	349.38 ± 0.76	351.84 ± 0.92	349.93 ± 0.99	0.769
T_p_ (°C)	368.56 ± 1.72	369.55 ± 1.77	367.39 ± 1.18	369.56 ± 0.47	369.00 ± 2.04	0.844
T_c_ (°C)	384.52 ± 2.99	389.50 ± 3.84	386.07 ± 2.65	385.51 ± 1.37	393.97 ± 3.66	0.242
T_c_–T_o_ (°C)	33.51 ± 1.34 ^b^	38.38 ± 2.33 ^ab^	36.69 ± 2.06 ^b^	33.68 ± 0.80 ^b^	44.05 ± 3.53 ^a^	0.040
ΔH (J g^−1^)	48.33 ± 1.03 ^b^	36.38 ± 2.53 ^bc^	61.31 ± 1.67 ^a^	29.25 ± 6.30 ^c^	63.94 ± 3.54 ^a^	0.003
ΣΔH (J g^−1^)	52.63 ± 4.70 ^ab^	46.14 ± 6.19 ^bc^	59.33 ± 4.17 ^ab^	29.25 ± 6.30 ^c^	68.16 ± 6.07 ^a^	0.020

Note: T_o_, onset temperature; T_p_, peak temperature; T_c_, conclusion temperature; T_c_–T_o_, temperature range; ΔH, transition enthalpy; ND, not detected. Data are expressed as mean ± standard error of mean (*n* = 3 per treatment). Differences between means were tested with Duncan’s multiple range test. Different superscripts in the same row indicate a significant difference (*p* < 0.05).

**Table 3:** Carapace elemental profiles (% of dry weight) of northern river terrapins reared with various background colors. The sampling represents the end of the twelve-week experiment.

Element	Background Color					*p*-Value
	Transparent	Green	Red	Blue	Black	
C	47.14 ± 0.44	47.74 ± 1.46	47.67 ± 0.36	48.96 ± 0.83	45.53 ± 0.49	0.134
O	32.79 ± 0.41 ^a^	32.26 ± 0.51 ^a^	31.81 ± 0.10 ^ab^	30.66 ± 0.36 ^b^	32.23 ± 0.57 ^a^	0.046
N	14.67 ± 0.87	12.46 ± 1.00	10.97 ± 0.41	12.63 ± 1.42	14.41 ± 0.47	0.084
Fe	5.98 ± 0.47	7.04 ± 1.67	8.11 ± 0.71	6.59 ± 1.22	7.16 ± 1.46	0.732
P	0.21 ± 0.01	0.38 ± 0.14	0.50 ± 0.09	0.40 ± 0.11	0.26 ± 0.07	0.267
Al	0.10 ± 0.01	0.14 ± 0.03	0.19 ± 0.06	0.11 ± 0.01	0.13 ± 0.01	0.352
Si	0.16 ± 0.01	0.23 ± 0.06	0.19 ± 0.02	0.26 ± 0.04	0.13 ± 0.01	0.125
Mg	0.11 ± 0.01	0.16 ± 0.02	0.13 ± 0.03	0.14 ± 0.02	0.10 ± 0.01	0.532
Na	0.18 ± 0.01	0.27 ± 0.06	0.23 ± 0.02	0.20 ± 0.01	0.13 ± 0.04	0.120
S	0.17 ± 0.04 ^b^	0.26 ± 0.01 ^ab^	0.35 ± 0.06 ^a^	0.34 ± 0.04 ^a^	0.18 ± 0.04 ^b^	0.028
Ca	0.12 ± 0.01 ^bc^	0.23 ± 0.05 ^abc^	0.26 ± 0.06 ^a^	0.25 ± 0.05 ^ab^	0.11 ± 0.01 ^c^	0.049

Data are expressed as mean ± standard error of mean (*n* = 3 per treatment). Differences between means were tested with Duncan’s multiple range test. Different superscripts in the same row indicate a significant difference (*p* < 0.05).

**Table 4:** Hematological parameters of northern river terrapins reared with various background colors. The sampling represents the end of the twelve-week experiment.

Hematological Parameter	Background Color					*p*-Value
	Transparent	Green	Red	Blue	Black	
RBC (×10^6^ cells μL^−1^)	0.29 ± 0.03 ^b^	0.35 ± 0.03 ^ab^	0.47 ± 0.09 ^a^	0.27 ± 0.04 ^b^	0.48 ± 0.04 ^a^	0.039
WBC (×10^3^ cells μL^−1^)	0.11 ± 0.01 ^b^	2.42 ± 0.58 ^a^	3.08 ± 1.35 ^a^	3.92 ± 0.57 ^a^	3.50 ± 0.21 ^a^	0.040
Hematocrit (%)	25.33 ± 1.76	22.67 ± 0.67	26.00 ± 2.31	22.33 ± 1.86	26.00 ± 1.15	0.365
MCV (mm^3^)	917.32 ± 147.30 ^a^	649.32 ± 48.37 ^bc^	582.65 ± 61.01^bc^	837.93 ± 41.69 ^ab^	549.34 ± 35.20 ^c^	0.029
Lymphocyte (%)	8.00 ± 1.00 ^b^	19.33 ± 0.67 ^a^	21.00 ± 1.00 ^a^	18.00 ± 2.89 ^a^	23.33 ± 2.91 ^a^	0.018
Azurophil (%)	80.00 ± 3.21 ^a^	71.33 ± 1.33 ^a^	76.67 ± 4.06 ^a^	74.33 ± 4.33 ^a^	60.33 ± 2.33 ^b^	0.014
Plasma protein (g%)	2.60 ± 0.12	1.67 ± 0.18	2.33 ± 0.66	1.87 ± 0.13	2.80 ± 0.20	0.148
BUN (mg dL^−1^)	31.85 ± 0.95	34.05 ± 1.35	34.83 ± 1.31	40.05 ± 7.65	30.43 ± 2.15	0.345
Creatinine (mg dL^−1^)	0.60 ± 0.04	0.59 ± 0.05	0.55 ± 0.02	0.56 ± 0.01	0.61 ± 0.01	0.758
ALP (U L^−1^)	212.50 ± 14.50	258.50 ± 29.50	200.50 ± 6.50	195.50 ± 12.50	260.00 ± 4.00	0.086
ALT (U L^−1^)	5.72 ± 0.29	8.89 ± 0.63	6.00 ± 0.95	7.36 ± 0.95	ND	0.106
Cortisol (μg dL^−1^)	ND	ND	ND	ND	ND	–

Note: RBC, red blood cells; WBC, white blood cells; MCV, mean corpuscular volume; BUN, blood urea nitrogen; ALP, alkaline phosphatase; ALT, alanine aminotransferase; ND, not detected (<5 U L^−1^ and <0.02 μg dL^−1^ for ALT and cortisol, respectively). Data are expressed as mean ± standard error of mean (*n* = 3 per treatment). Differences between means were tested with Duncan’s multiple range test. Different superscripts in the same row indicate a significant difference (*p* < 0.05).
